# Species-Level Analysis of DNA Sequence Data from the NIH Human Microbiome Project

**DOI:** 10.1371/journal.pone.0047075

**Published:** 2012-10-10

**Authors:** Sean Conlan, Heidi H. Kong, Julia A. Segre

**Affiliations:** 1 Genetics and Molecular Biology Branch, National Human Genome Research Institute, National Institutes of Health, Bethesda, Maryland, United States of America; 2 Dermatology Branch, Center for Cancer Research, National Cancer Institute, National Institutes of Health, Bethesda, Maryland, United States of America; Baylor College of Medicine, United States of America

## Abstract

**Background:**

Outbreaks of antibiotic-resistant bacterial infections emphasize the importance of surveillance of potentially pathogenic bacteria. Genomic sequencing of clinical microbiological specimens expands our capacity to study cultivable, fastidious and uncultivable members of the bacterial community. Herein, we compared the primary data collected by the NIH’s Human Microbiome Project (HMP) with published epidemiological surveillance data of *Staphylococcus aureus*.

**Methods:**

The HMP’s initial dataset contained microbial survey data from five body regions (skin, nares, oral cavity, gut and vagina) of 242 healthy volunteers. A significant component of the HMP dataset was deep sequencing of the 16S ribosomal RNA gene, which contains variable regions enabling taxonomic classification. Since species-level identification is essential in clinical microbiology, we built a reference database and used phylogenetic placement followed by most recent common ancestor classification to look at the species distribution for *Staphylococcus*, *Klebsiella* and *Enterococcus*.

**Main Results:**

We show that selecting the accurate region of the 16S rRNA gene to sequence is analogous to carefully selecting culture conditions to distinguish closely related bacterial species. Analysis of the HMP data showed that *Staphylococcus aureus* was present in the nares of 36% of healthy volunteers, consistent with culture-based epidemiological data. *Klebsiella pneumoniae* and *Enterococcus faecalis* were found less frequently, but across many habitats.

**Conclusions:**

This work demonstrates that large 16S rRNA survey studies can be used to support epidemiological goals in the context of an increasing awareness that microbes flourish and compete within a larger bacterial community. This study demonstrates how genomic techniques and information could be critically important to trace microbial evolution and implement hospital infection control.

## Introduction

Epidemiology is a cornerstone of public health research. Tracking the prevalence and spread of bacterial pathogens in the population is central to the formulation of healthcare policy. For example, the Centers for Disease Control's Active Bacterial Core surveillance program focuses on the incidence of invasive disease from a number of pathogenic organisms. Similarly, hospitals have local surveillance protocols to track the incidence of potential pathogens such as *Staphylococcus aureus*, *Klebsiella pneumoniae* and *Enterococcus spp.*, relying on classical culturing and typing of organisms in hospital or reference laboratories. These reliable and widely available methods focus on individual known cultivable organisms. It is increasingly clear that microbes flourish and compete within larger bacterial communities. Epidemiological studies focused on tracking the incidence of antibiotic-resistant bacteria benefit from complementary genomics studies of microbial communities *in toto* to investigate quantitative changes in species-level prevalence, and to understand bacterial population structure.

Metagenomics is the direct sequencing of complete microbial communities, analyzing total genomic content. Metagenomics provides an opportunity to survey both fastidious and easily cultivatable bacteria in the global microbial community. The number of published metagenomic studies has increased with the advent of new high-throughput DNA sequencing instruments. Recent projects, including MetaHIT [1] and NIH’s Human Microbiome Project (HMP) [2]–[4], have produced terabases (10^12^) of metagenomic sequence data. While not previously analyzed in the context of microbial surveillance, the data produced by these studies have presented a unique opportunity to measure the abundance of a wide variety of potential pathogens in different populations. However, genomic sequencing analyses struggle with the complication of identifying sequences beyond the genus level to the species level. For example, clinically and biochemically distinct bacterial species such as *Staphylococcus epidermidis* ATCC12228 and *Staphylococcus aureus* N315 have nearly identical 16S rRNA gene sequences, differing at only a handful of positions. Nucleotide (nt) differences between 16S rRNA genes of closely related organisms are not distributed evenly across the gene, but rather are clustered in variable regions, reflecting the three-dimensional structure of the rRNA. Here, we demonstrate that selecting the correct region of the 16S rRNA gene to sequence is comparable to selecting the appropriate culture conditions to distinguish closely related bacterial species. Published metagenomics and cultivation studies provide complementary information.

In this study, we analyzed NIH’s HMP dataset, which used deep sequencing of samples from hundreds of normal volunteers to characterize the microbial communities of the oral cavity, skin, nares, gastrointestinal tract, and urogenital tract. In many cases the five “body sites” were made up of a number of distinct sites, i.e. four distinct skin sampling sites. As of May 2010, the HMP produced nearly three terabases of 16S rRNA sequencing data, which has been deposited in a public database (NCBI Bioproject 48333). These data were derived from a population of carefully screened healthy volunteers with up to two clinic visits. We studied three representative human-associated bacteria with potential for pathogenicity and acquisition of antibiotic-resistance. We used phylogenetic placement and classification to assign short-read (∼300 nt) 16S rRNA sequences to the species-level based on a curated database and measured the carriage rate of the pathogens *S. aureus, K. pneumoniae* and *E. faecalis*.

## Results

### Construction of a 16S rRNA Reference Database

The full-length bacterial 16S rRNA sequence is approximately 1500 nt long. Species within a genus typically differ by 1–3%, corresponding to a total of 15–45 base pair (bp) differences. While a small number of high-confidence nucleotide differences between sequences can provide sufficient resolution to distinguish closely related species, genomic technology has moved toward high-throughput sequencing of short fragments in the 100 to 400 nt range [5]. These shorter sequences produced by newer technologies are extremely cost-effective to study population dynamics, but create a challenge if the study needs to classify sequences to the species level. To examine the ability to differentiate among different species in the HMP dataset, we took the genus *Staphylococcus* as an example. We considered two possibilities for building a high-confidence reference dataset: (1) utilizing a greater number of sequences available from public databases or (2) compiling a group of select, highly curated sequences.

From a genomics perspective, gathering the largest number of possible sequences is the most comprehensive strategy to construct a database. To begin our analysis, we examined the annotation quality of *Staphylococcal* sequences within the Ribosomal Database Project (RDP) [6], Greengenes [7] and SILVA [8] databases. Sequences were filtered using each database's web portal to include only high-quality data. Initially, we selected only sequences from the *Staphylococcus* taxon for further analysis. Searching the annotations within this data set, we found many sequences that had non-specific labels (e.g., “Uncultured bacterium”), were missing a species designation, or were labeled as other genera. Filtering each database for only those sequences labeled with both a genus and a species removed 28–83% of sequences, reflecting differences in annotation quality across databases. These filtered sequences were placed in a phylogenetic tree to determine whether the species labels were consistent with the tree topology. All three databases contained sequences that were likely mislabeled at the species-level based on their position within otherwise monophyletic clades of sequences (e.g., EF528274, EU664599, AM945662). When misannotated ‘reference’ sequences are included in a most recent common ancestor analysis, they have the effect of either forcing classifications to higher taxonomic levels or generating inaccurate assignments [9]. After identifying annotation errors in our initial analysis, we considered the second option of creating a curated database. Rather than subtracting mislabeled sequences from these large datasets, we settled on a hybrid approach and built a curated database of 40 *Staphylococcal* 16S rRNA sequences based on the non-redundant union of complete genomes at NCBI (n = 71) and type species at RDP (n = 54). Since this resulted in a single sequence representative for some species, the trusted sequences were use to “recruit” additional sequences into sparsely populated species (see materials and methods).

### Accurate Species Labeling is Dependent on the Choice of Region

**Figure 1:**
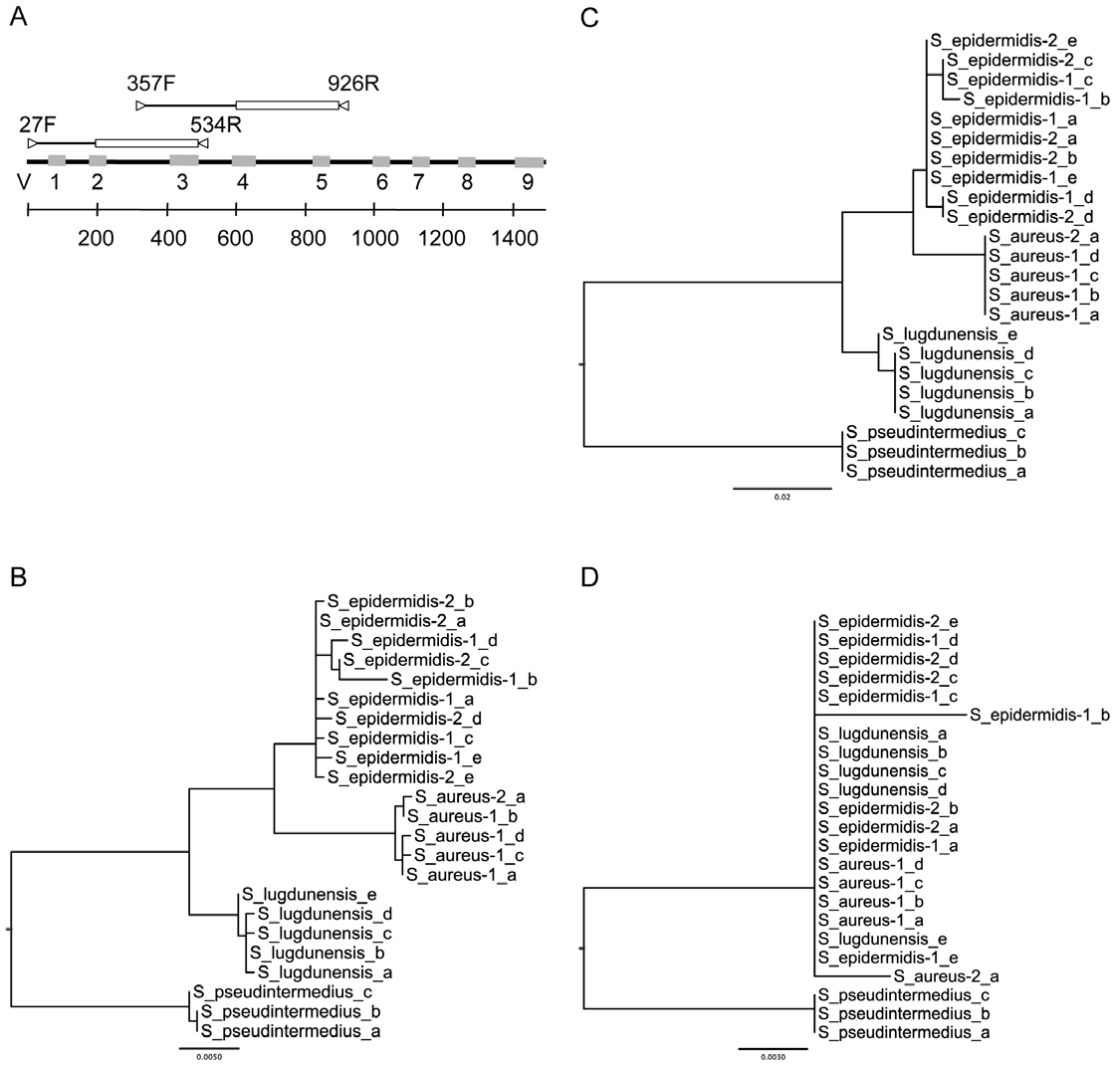
Maximum-likelihood phylogenetic trees of 16S rRNA variable regions for selected *Staphylococcus spp.* (**A**) Diagram of the 16S rRNA gene with variable regions 1–9 indicated in gray. Amplification primers are shown as triangles. The 300 nt subsequences used to construct the trees are boxed. The phylogenetic trees shown were generated for the Staphylococcus species in the reference database and are based on the (**B**) full-length sequence (**C**) V1-3 region and (**D**) V3-5 region. Four *staphylococcal* species are shown on the tree for clarity. However, 40 *staphylococcal* species such as *S. hominis, S. capitas, S. haemolyticus, S. saprophyticus, S. carnosus* were included in the full analysis. Full strain names are: S_aureus-1 =  *Staphylococcus aureus* USA300 TCH1516 (NC_010079); S_aureus-2 =  *Staphylococcus aureus* USA300 FPR3757 (NC_007793); S_epidermidis-1 =  *Staphylococcus epidermidis* ATCC 12228 (NC_004461); S_epidermidis-2 =  *Staphylococcus epidermidis* RP62A (NC_002976); S_lugdunensis  =  *Staphylococcus lugdunensis* HKU09-01 (NC_013893); S_pseudintermedius  =  *Staphylococcus pseudintermedius* HKU10-03 (NC_014925). Lower case letters indicate independent copies of the 16S rRNA operon.

After building a high-quality reference database, we examined regions of the 16S rRNA gene that would provide sufficient power for species-level identification. 454 XLR pyrosequencing reads, which are typically 400 nt in length but often quality-trimmed to ∼300 nt, are commonly used to capture variable regions 1–3 (V1-3) or 3–5 (V3-5) (Figure 1A) [10]. These regions are sequenced by many projects because of the flanking universal primer binding sites and the read-length of the platform. We extracted the V1-3 and V3-5 regions from the reference alignment for *Staphylococcus* to compare their discriminatory power at the species-level. The *Staphylococcal* V1-3 region recapitulated the species-level discrimination achieved with full-length sequences (Figure 1B) and thus could be used to differentiate species (Figure 1C). In contrast, V3-5 regions from different *Staphylococci* were often identical, resulting in a largely unstructured phylogenetic tree (Figure 1D). As an example, 16S rDNA sequences from *S. aureus* N315 and *S. epidermidis* ATCC12228 were 98% identical across their entire length. The 300 nt V1-3 fragments from these two species were 97% identical with 10 base pair differences, while the V3-5 region was 100% identical with no discriminatory power. We, therefore, limited further analysis to the V1-3 sequencing data for *Staphylococcal* species. Our results agreed with analysis performed on the larger HMP dataset, which confirmed that V1-3 region had higher resolving power [4].

### Sampling Depth and Completeness

We used HMP’s high quality16S rDNA data set for our analysis (see Materials and Methods). These data were released publicly in May 2010 as a test set to develop and standardize analytical techniques. We included in our analysis clinical samples with ≥1000 sequences after filtering. The median read count per sample was 6,332 with a maximum read count of 159,482. Although the HMP study design included 18 body sites for women and 15 for men, the median number of sites passing our filtering criteria was 13 per subject. The May 2010 data release provides only partial data for subjects sequenced for a second visit, limiting the ability to draw conclusions about patterns over time.

### Carriage of Staphylococci in a Healthy Population

HMP 16S rRNA V1-3 sequence data classified as genus *Staphylococcus* were further classified to the species level using a most recent common ancestor analysis of the phylogenetic placements generated by pplacer [11]. Briefly, pplacer takes as input a fasta file of aligned reads and a phylogenetic reference package made up of a sequence alignment, phylogenetic tree, taxonomy outline and phylogenetic model. The input reads are placed into the fixed reference phylogeny using maximum likelihood criteria. The collections of placements are then used as input to the classify command in the guppy tool (distributed with pplacer) to produce most recent common ancestor classifications. Species-level assignments were made where the classification likelihood was greater than a package-dependent threshold (see material and methods). Using pplacer and a classification likelihood cutoff of 0.85, we were able to assign 76% of the *Staphylococcus* sequences to species-level taxa.

**Figure 2:**
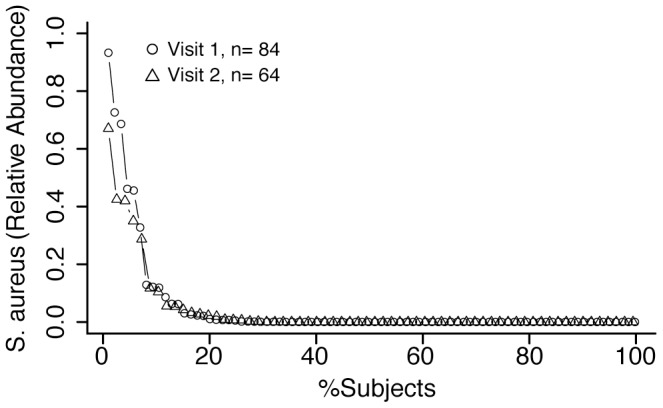
Distribution of *staphylococci* in the nares. The relative abundance of *S. aureus* in the nares for patients’ clinic visit. Each symbol represents the relative abundance of *S. aureus* in a single subject.

**Figure 3:**
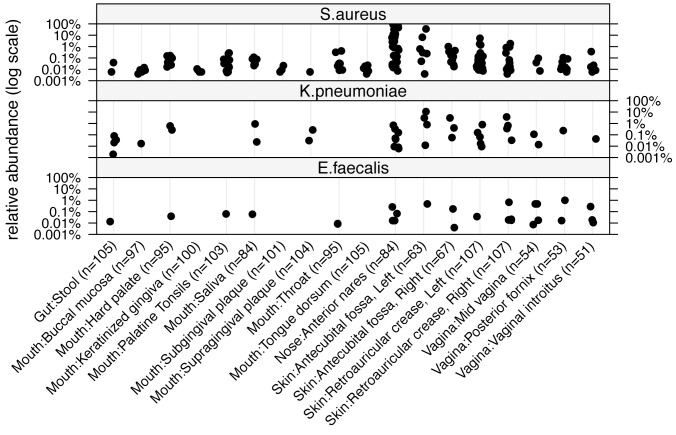
Relative abundance of *S. aureus*, *K. pneumoniae* and *E. faecalis* across body sites and individuals. Each point represents the relative abundance of an organism for an individual's first sampling visit. Relative abundances are shown on a log scale. Samples along the x-axis are described by general and specific body site.

Of sequences classified as the genus *Staphylococcus*, *S. epidermidis* was the dominant species. Particularly in the nares and retroauricular crease, *S. epidermidis* comprises >50% of the *Staphylococcal* population. *S. aureus* was less common in these sites, but found in several subjects at high relative abundance. Since the anterior nares are a clinically relevant site for screening *S. aureus* carriage, we analyzed data for anterior nares to determine the fraction of the population harboring *S. aureus* (Figure 2) and found that 36% of subjects were positive for *S. aureus* at the first sampling visit and 31% at visit two. We also detected *S. aureus* in samples from the antecubital crease and retroauricular crease, with and without co-occurrence in the nares. *S. aureus* was found at low relative abundance (<1%) in body sites associated with the oral cavity, stool and the vagina (Figure 3).

### *Carriage of* Klebsiella Pneumoniae *and* Enterococcus Faecalis

We also analyzed two species of Gram-negative bacteria, *Klebsiella pneumoniae* and *Enterococcus faecalis*. These bacteria are part of the normal human flora but are also associated with hospital-acquired infections and are becoming increasingly resistant to antibiotics. Of particular concern amongst immune-compromised patients are infections of carbapenemase-resistant *Klebsiella pneumoniae* and vancomycin-resistant *Enterococcus faecium* and *faecalis.* In order to characterize the species distributions for these two genera in the HMP data, we built phylogenetic reference packages for each. As above, we identified sequences annotated as belonging to the *Klebsiella* and *Enterococcus* taxa that were clearly misannotated. For instance, HQ670758 is annotated as a *K. pneumoniae* sequence but is 99% identical to *Pseudomonas aeruginosa* sequences. Sequences annotated as *Enterococcus* were found to match the *Streptococcus* and *Lactobacillus* genera (AB232954, HQ259240). We built reference packages for the *Klebsiella* and *Enterococcus* genera and used phylogenetic placement and classification to make species-level assignments.

We found *K. pneumoniae* in 3.8% of healthy volunteer stool samples and in 9.5% of samples from the nares. Sites from the skin, mouth and vagina were also colonized but typically at much lower rates than was seem for *S. aureus* (Figure 3). *E. faecalis* was found at a low relative abundance (<1%) sporadically across body sites and individuals (Figure 3). *E. faecium* wasn’t found at appreciable levels in any clinical sample. We considered the rates of co-colonization for a subset of individuals with data for at least one site in each major body site: mouth, gut, nares, skin and vagina (n = 118). Overall, 78 individuals (66%) were colonized with one or more of the organisms shown in Figure 3 at one or more sites. Rates of colonization with one, two or three organisms were 42%, 20% and 5% respectively. While most subjects were colonized at one or two body sites, some individuals were positive for one or more organisms across three, four or five body sites.

## Discussion

We have demonstrated that, analogous to selecting the correct media conditions in a microbiology lab, a well-designed sequencing strategy is essential in determining the utility of microbial genomics for pathogen surveillance and species-level assignments. For the genus *Staphylococcus*, the 5' end of the 16S rRNA gene that includes V1-3 discriminates between many species and is superior to the V3-5 region. The discriminatory power of the V1-3 region has been shown previously for *Helicobacter pylori* [12]. An early study examined nucleotides in the V1-3 region to discriminate within genera such as *Staphylococcus*, *Streptococcus*, *Enterococcus* [13]. It is worth noting that there is no single “best” variable region for all bacteria, despite the availability of broadly informative primer sets [14]. For instance, a detailed investigation of primer pairs for analysis of the foregut microbiome found the V3-5 region to be the most useful for classifying 16S rRNA genes [15]. In addition, while *Staphylococcus*, *Klebsiella* and *Enterococcus* could be classified using V1-3 data, species-level determinations for *Pseudomonas* and *Neisseria* were less robust using V1-3 data.

These genus-dependent and variable region-dependent effects led us to use “leave one out” classification and classification of extracted 16S rDNA variable regions as a guide in setting a minimum confidence level for accepting species-level classifications from each reference package (see Materials and Methods). In addition, classifications of *S. aureus*, *K. pneumonia* and *E. faecalis* were examined manually to identify instances where related sequences might be misclassified resulting in false positives. For instance, *S. simiae* is difficult or impossible to separate from *S. aureus* based on V1-3 data. *S. simiae* is associated with squirrel monkeys so it is unlikely to be found in the healthy HMP study population but this highlights a potential pitfall of this approach where zoonotic diseases or truly novel species are suspected. Sequence length and read accuracy, fundamental components of this and other genomics-based studies, are also important for correct species assignment.

Metagenomic sequencing provides an opportunity to study global communities including even fastidious bacteria, yet this methodology has its own set of limitations including systematic bias and random noise [16]. For instance, we were often limited by sparse coverage of species in the public databases and errors in taxonomic annotation. Our analysis sought to determine the presence or absence of one or more organisms in a large dataset, allowing us to avoid issues often plaguing ecological studies including overestimation of diversity. However, our findings likely represent the lower limit for carriage rates. Low abundance organisms will require deeper sequencing for detection, particularly in samples with high bacterial loads such as stool. Of note, even culture-based methods have demonstrated Type II errors, including single-plate culturing of *S. pyogenes* [17]. Metagenomic sequencing provides information complementary to culture-based measurements.

The Human Microbiome Project has sought to characterize the normal commensal human flora and to provide a baseline for interpreting perturbations associated from disease states. We have shown that the HMP provides valuable data regarding the carriage of potentially pathogenic organisms in this healthy population. We analyzed the read data to identify carriers of *S. aureus* and identified one-third of individuals as carriers, comparable to what has been reported previously in culture-based studies [18]–[19]. Public health concerns persist regarding the carriage of methicillin-resistant *S. aureus* (MRSA), estimated at 1.5% of the population [20]. Analysis of 16S rRNA data alone cannot determine MRSA carriage in the HMP population. However, our preliminary analysis of whole genome shotgun data indicated the presence of the *mecA* gene, which encodes methicillin-resistance, in a small percentage of these samples. Although these methods could not distinguish whether the *mecA* gene was associated with *S. epidermidis* or *S. aureus*, resistance genes can be transferred from the former to the later.

Understanding the prevalence and distribution of commensals with the potential to become antibiotic resistant pathogens in a healthy population can inform refinement of methods used for surveillance of potential pathogens. *K. pneumoniae* was found less frequently in stool (3.8%) than has been reported previously (13.1%) [21], possibly due to the limited sampling depth or other biases in sequencing. In a study of a *K. pneumoniae* outbreak in 2001, colonization rates were higher for the trachea and nares compared to the gut [22]. However, extensive surveillance is essential to detect antibiotic-resistant *K. pneumoniae* isolates in patients who might be colonized without showing signs of infection, such as during a hospital outbreak [23]. 16S rDNA sequencing holds potential to more sensitively identify patients colonized with *K. pneumoniae* for whom further testing should be performed to determine if the organisms harbor antibiotic resistance.

While *K. pneumoniae* is known to inhabit a variety of niches, including the gut, mouth and skin, *E. faecalis* is generally considered to be a gut commensal. We detected *E. faecalis* sporadically in samples from the mouth, nares, skin and vagina. We found that 6 of 58 women with sequencing data for one of the three vaginal sites were positive for *E. faecalis*. While these subjects were healthy at the time of sampling, *E. faecalis* is known to cause urinary tract infections. Once the complete HMP dataset is available, the persistence of colonization over time in this population can be analyzed. In addition, it will be possible to measure associations between clinical metadata (e.g., smoking status) and abundance of members of the human microbiome.

While culture-based studies will continue to be a critical component of bacterial pathogen surveillance, genomic-based approaches provide a powerful complementary technology. For instance, sequencing is valuable for detecting multiple organisms in a single sample, presence of fastidious organisms, and organisms difficult to cultivate due to ongoing antibiotic treatments. Furthermore, full metagenomic approaches provide the ability to not only identify the organisms that are present in a sample, possibly to the subspecies or strain level [24], but also to detect the presence of antibiotic resistance genes and virulence factors. Metagenomics data provide a tool for understanding the spread of multidrug resistance in the population and provides clinicians with additional data for planning long-term surveillance and treatment strategies.

## Methods

### Reference Database and Taxonomic Reference Packages

Analysis of taxonomic annotation accuracy was based on data downloaded from three widely used public databases including: Ribosomal Database Project (RDP, Release 10, Update 26), Greengenes (Release 3/30/2011), Silva (Release 104) and NCBI. A high-quality “trusted” 16S rRNA reference database was built for our analysis using NCBI rRNA records extracted from RefSeq genomes (as of April 2012) and RDP type species sequences (Release 10, Update 26). The combined sequences were merged and further processed using MOTHUR (v1.22.0), a suite of microbial analysis tools [25]. Specifically, sequences were aligned to the 7,682 column-wide Greengenes reference alignment (as of 10/2/2011) to account for the architecture of the 16S rRNA gene [7], screened to remove short or ambiguous sequences and then clustered to identify unique sequences. Phylogenetic trees were built using PhyML (v3.0) with the GTR model [26]. For variable region trees, the positions of the sequencing primers 534R and 926R (see below) were used to determine the left-hand (5') trimming positions for V1-3 and V3-5, respectively. Right-hand (3') trimming positions were set at a fixed distance of 300 nt upstream of the sequencing primer to adjust for 454 sequencing reads which capture only part of the region.

Taxonomic reference packages for *Staphylococcus*, *Klebsiella* and *Enterococcus* were built using the taxtastic suite of tools (taxit v0.3.1; https://github.com/fhcrc/taxtastic) [11]. Specifically, sequences for each genus were extracted from the trusted set of sequences described above. Since, classification using a most recent common ancestor approach requires a given taxa to have several sequence representatives, species with fewer than five trusted sequences were supplemented with additional sequences where possible. Sequences were recruited from the non-type sequence set using BLASTN with the trusted sequences as queries. Sequences were added to the package if they met the following three criteria: shared species annotation with a trusted sequence, > = 97% identity over 1300 nt to a trusted sequence, not identical to any trusted sequence. The trusted sequences and any sequences recruited by them were aligned and a phylogenetic tree was built as described above. These data were used to generate a taxonomic package using the taxtastic suite. As a final refinement step, the rppr tool (distributed with pplacer) was used to identify leaves of the tree that didn’t agree with their taxonomic assignment [27]. These sequences were removed and a final package was built. The reference packages used in this manuscript are available from the authors by request.

Each reference package is dependent upon a phylogenetic tree that is generated from available sequence data. This tree, combined with the taxonomic outline and the region of 16S rDNA being sequenced ultimately determines how accurate a package will be for speciation. Each package was tested to determine a likelihood cutoff. First, the package was subjected to leave-one-out analysis where all sequences for a species were removed from the package and then classified against the remaining species. This provides an estimate of the false positive rate since any classification with a likelihood better than the threshold is a false positive. In addition, the reference alignment was trimmed to include only the region covered by the 454 PCR amplicon and classified, giving an approximation for the true positive rate. This analysis also allows a comparison of variable regions and in all cases the V1-3 region gave a higher true positive rate than the V3-5 region for each of the three genera examined. A likelihood cutoff was selected for each package such that accuracy (True Positives + True Negatives) was maximized. In addition, to setting a likelihood threshold, species of particular interest in this study were examined manually to identify instances where sequences might be misclassified resulting in false positives.

### HMP Dataset

The 16S rRNA gene is present in all bacteria/archaea and contains both variable regions, enabling taxonomic classification, and conserved regions, which serve as binding sites for PCR primers. The HMP dataset contained 16S rRNA gene sequences from 242 healthy volunteers surveyed at 15 or 18 individual body sites across the oral cavity, skin, nares, gastrointestinal tract, and urogenital tract. Details of the HMP Clinical protocol are described at the DACC (http://www.hmpdacc.org) [2]. Briefly, DNA was prepared directly from clinical specimens (typically a swab of the body site). The 16S rRNA gene was amplified directly from the DNA and then sequenced on the Roche/454 XLR instrument to yield sequence lengths of 300–500 nt. Of the nine variable (V) regions, V1-3 and V3-5 were selected by the HMP and many previous sequencing projects as containing the proper attributes of conserved flanking regions for primer design and variable regions for taxonomic assessment. The forward and reverse primers used were: 8F - AGA GTT TGA TCC TGG CTC AG and 534R - ATT ACC GCG GCT GCT GG for the V1-3 region; 521F - CCT ACG GGA GGC AGC AG and 926R - CCG TCA ATT CMT TTR AGT for the V3-5 region. Access to data from the HMP Core Microbiome Sampling Protocol was authorized through dbGaP DAC request #7655-2. 16S rRNA sequences from the High Quality processing pipeline [4], [28] longer than 320 nucleotides (nt) were obtained from the HMP Data Analysis and Coordination Center (http://www.hmpdacc.org) (release date: May 2010). Reads longer than 320 nt were trimmed to obtain a uniform dataset. Metadata for each sample was obtained from dbGaP (http://www.ncbi.nlm.nih.gov/gap) and converted to tabular form using custom scripts (Curtis Huttenhower, personal communication). The sequences were stored along with metadata in a MySQL database for further analysis. When analyzing sequences from a single site for the presence or absence of an organism, one read was considered sufficient to assign that sample as positive. This interpretation is the most analogous to culture based surveys, which are very sensitive to low numbers of organisms.

### Speciation Pipeline

As part of the HMP’s High Quality processing pipeline, each 16S rRNA gene sequence was assigned a taxonomic classification by the RDP naïve Bayesian classifier [6]. We extracted those sequences assigned to *Staphylococcus*, *Klebsiella* or *Enterococcus* and compared them to sequences from the curated reference packages described above. Sequences were placed in a fixed phylogenetic reference tree using the pplacer algorithm (v1.1.alpha13-0-g1ec7786; http://matsen.fhcrc.org/pplacer) [11] with “–keep-at-most 100–max-pitches 100”. Taxonomic classifications were generated using a most recent common ancestor approach implemented in the guppy program (guppy is distributed with pplacer). Species-level assignments were accepted when the likelihood of the classification was greater than the per-package cutoff determined above (*Staphylococcus*: 0.85, *Klebsiella*: 0.90, *Enterococcus*: 0.65).
